# A Physical Therapy Rehabilitative Approach in Improving Activities of Daily Living in a Patient With Morel-Lavallée Syndrome: A Case Report

**DOI:** 10.7759/cureus.29523

**Published:** 2022-09-24

**Authors:** Divya M Badjate, Deepak Jain, Pratik Phansopkar, Om C Wadhokar

**Affiliations:** 1 Department of Musculoskeletal Physiotherapy, Ravi Nair Physiotherapy College, Datta Meghe Institute of Medical Sciences, Wardha, IND; 2 Department of Physiotherapy, Ravi Nair Physiotherapy College, Datta Meghe Institute of Medical Sciences, Wardha, IND; 3 Department of Musculoskeletal, Ravi Nair Physiotherapy College, Datta Meghe Institute of Medical Sciences, Wardha, IND

**Keywords:** clinical case report, orthopaedic surgery, rehabilitation, physical therapy, morel – lavallee syndrome

## Abstract

A limited soft tissue injury known as Morel-Lavallée syndrome is brought on by the violent segregation of the dermis and subcutaneous tissue layer. Shear injury causes perforating arteries and lymphatics to burst, potentially releasing serosanguinous fluid, blood, and necrotic fat into the area. Morel-Lavallée lesions (MLL) can be accompanied by pelvic or acetabular fractures or by blunt trauma without a fracture. MLL is distinguished by enlargement, tightness, and tenderness of tissue. The skin around the lesion is frequently associated with diminished sensory perception. Plain radiography, USG, CT scan, and MRI are some of the imaging modalities that can be utilized. MLLs have a distinct location in the US, anterior to the muscle layer and posterior to the hypodermis. Morel-Lavallée lesions are frequently associated with complications as a result of delayed or inappropriate diagnosis. Surgical drainage and debridement are the standard surgical treatments for the lesion. Physiotherapy rehabilitation helps in reducing pain and regaining functional activities after the syndrome. This documentation includes the case of a 55-year-old male patient who had complaints of difficulty in moving their left leg and inability to walk. The patient was diagnosed with Morel-Lavallée syndrome left thigh and was advised physiotherapy. This study found that by employing strengthening exercises and other physical therapy interventions, after four weeks of therapy, joint degree of movement, muscular strength, and daily functioning, as well as cardiovascular and pulmonary capabilities all significantly improved.

## Introduction

A limited soft tissue injury known as Morel-Lavallée syndrome is brought on by the violent segregation of the dermis and subcutaneous tissue layer. Perforating arteries and lymphatics break as a result of sheer damage, potentially discharging serosanguinous fluid, blood, and necrotic fat into the region [1]. Although motor vehicle accidents are the most frequent reason, falls and sports-related injuries make up a large portion of cases of low-grade blunt force trauma [2]. Morel-Lavallée lesions (MLL) can be accompanied by fractures in the pelvis or acetabulum or by blunt trauma without any break in the bone. Trochanteric and proximal thigh areas are the most probable sites for Morel-Lavallée lesions [1]. MLL is characterized by enlargement, tightness, and tenderness of tissue, as well as ecchymosis, erythema, and skin abrasion. The existence of fluctuation within the lesion is an essential clinical characteristic [3]. The reduced cutaneous sensation is frequently linked with the skin over the degloving area [4]. Secondary cutaneous alterations such as dryness and cracking, discoloration, and even open necrosis may occur below the surface [2]. A meticulous six-stage imaging-based classification system for Morel- Lavallée lesions was established by Mellado and Bencardino in 2005. It is based on the lesion's shape, the signal intensity on T1 and T2 weighted images, the presence of a fibrous capsule, contrast enhancement, and the development of a sinus tract capsule [5].

Plain radiography, ultrasound, CT scans, & MRI are some of the imaging modalities that may be used to detect and characterize Morel-Lavallée lesions [2]. A nonspecific soft tissue mass on plain radiography may signal the need for additional diagnostic tests or a possible underlying fracture. MLL manifests as a well-defined, encased fluid collection that occasionally reveals fluid levels on CT scans due to cellular blood component sedimentation. The use of ultrasonography to confirm the existence of a suspected MLL has proven to be successful. MLLs have a distinct location in the US, which is anterior to the muscle layer and posterior to the hypodermis. The importance of MRI in the diagnosis of MLLs cannot be overstated. Their appearance is mostly determined by their age and the number of contents present within the lesion [6]. A Morel-Lavallée lesion must be recognized & treated as soon as feasible since distracting injuries in the polytraumatized patient might culminate in a missing or delayed diagnosis. Problems are frequently accompanied by Morel-Lavallée lesions as a result of the delayed or imprecise diagnosis. Untreated sores might proliferate & cause pressure necrosis of the underlying skin. Large areas of skin disintegration, as well as underlying problems, may ensue from this [7]. Compression banding, aspiration and incision, and evacuation with or without sclerotherapy may be used to treat an MLL, depending on the stage of the lesion. For acute instances with closed or no underlying bone fracture, compression banding can be performed [8]. Surgery is indicated if conservative therapy is failing. Standard surgical treatments for the lesion entail surgical drainage and debridement [9]. Physiotherapy rehabilitation helps in reducing pain and regaining functional activities after the syndrome.

## Case presentation

Patient information

A 55-year-old male patient who stated facing difficulties lifting his left leg & being unable to walk for a week was sent to physiotherapy. He was involved in a car accident and suffered injuries, such as superficial and deep cuts to his head, left shoulder, left thigh, and left knee. The pain was aggravated by movements and relieved by rest and medication. The patient was brought to a multi-specialty hospital where primary management and investigations such as an X-ray pelvis with both hips, USG abdomen, and pelvis, and CECT abdomen were done after 22 hours following the injury, which revealed loss of differentiation of plane and soft tissue, break in the continuity of soft tissue; mild splenomegaly; changes of cystitis, respectively and he was diagnosed as a case of Morel- Lavallée syndrome left thigh. He has started medications for the same and was advised physiotherapy. He has had Diabetes Mellitus type II for 15 years. Five years back patient had a road traffic accident by falling from a bike and sustaining an injury to his left ankle for which he was managed with a below-knee slab.

Clinical findings

**Figure 1:**
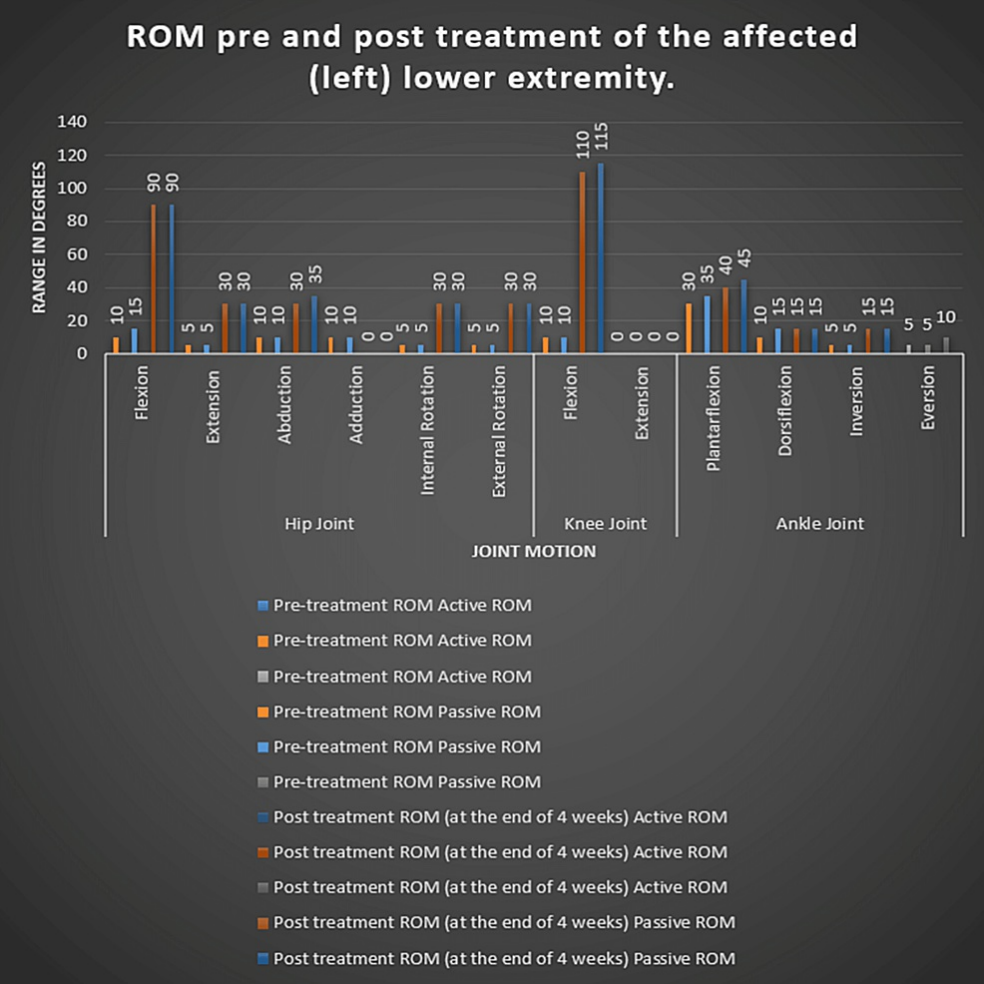
Range of motion pre- and post-treatment of the affected (left) lower extremity. The X-axis indicates joint motions of the lower extremity. The Y-axis indicates the range of motion in degree ROM: Range of motion

After acquiring the patient's signed agreement, the examination protocol was detailed to him. He was alert and well aware of time, location, & people while being examined. His vital signs were stable, and he showed no symptoms of cyanosis, icterus, clubbing, or edema. The patient was assessed while laying supine, & bandages could be seen concealing the patient's left lower abdomen and leg. The area that was impacted has edema. When the region was palpated, the temperature there rose, and there was grade 3 soreness there. Hip motions were unpleasant, and Figure 1 has already stated the affected lower extremity's range of motion. Based on the grading of resisted muscular contractions, muscle strength was assessed and is listed in Table 1.

**Table 1:** Resisted isometric contraction testing for muscles of the affected extremity

Muscles	Hip flexors	Hip extensors	Hip abductors	Hip adductors	Hip internal rotators	Hip external rotators	Knee flexors	Knee extensors	Ankle plantar flexors	Ankle dorsiflexors
Grading										
Weak and painful contraction	✔️	✔️	✔️	✔️	✔️	✔️				
Weak and painless contraction										
Strong and painful contraction							✔️	✔️		
Strong and painless contraction									✔️	✔️

All of the lower extremities' feelings were present throughout the neurological evaluation.

Timeline

The incident took place on 22 November 2021. On the next day, investigations such as an X-ray of the pelvis, left knee, USG abdomen and pelvis, CECT abdomen, USG thorax, and blood tests were done. Physiotherapy rehabilitation was started a week later.

Diagnostic assessment

**Figure 2:**
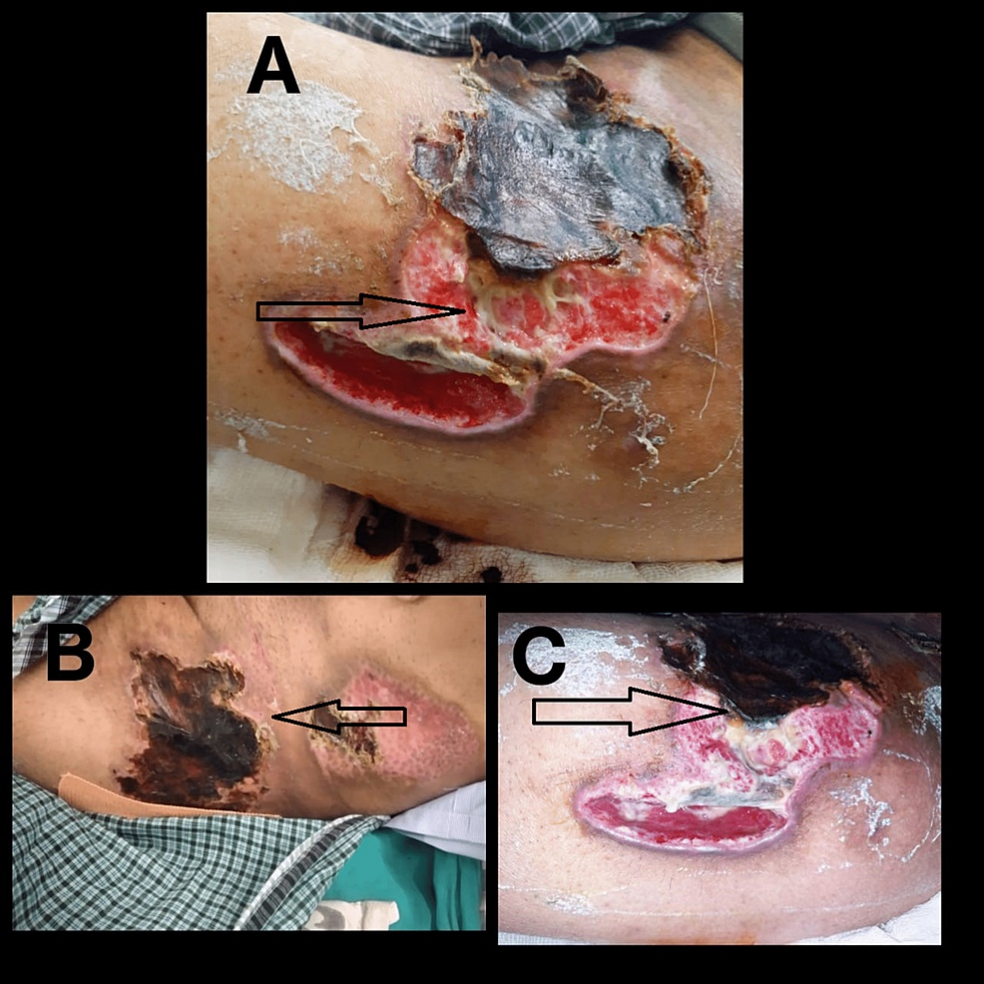
2A shows the lesion at the left anterior thigh on day three of injury. 2B shows the lesion at the left gluteal region on day three of injury. 2C shows the lesion at the left anterior thigh a month after injury.

The clinical images of the lesion are shown in Figure 2.

Diagnosis

The results revealed a Morel-Lavallée lesion of the left thigh.

Therapeutic intervention

**Figure 3:**
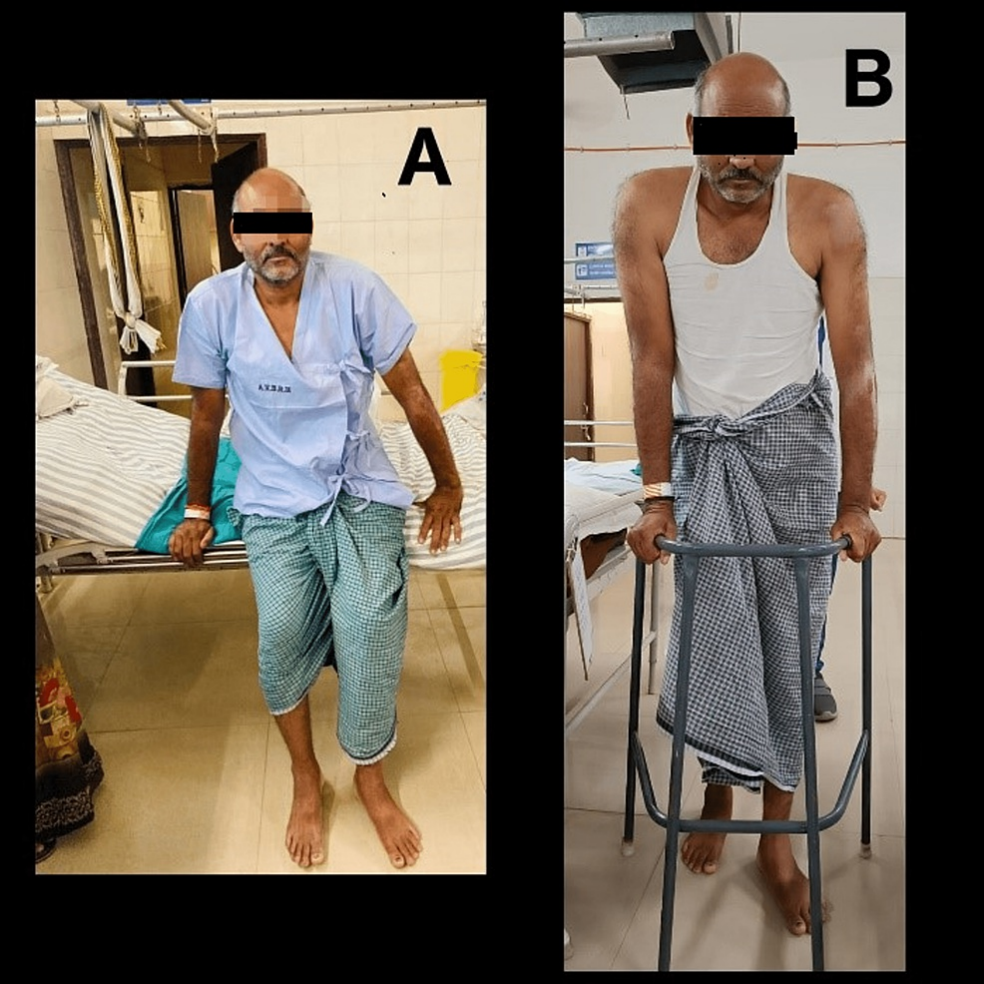
3A: Patient sitting at the edge of the bed. 3B: Patient walking with the help of a walker

Physiotherapy rehab was started after a week from the day of the injury, and the patient was treated for four weeks (Figure 3).

Short-term goals: It is aimed at educating the patient about the condition, alleviating pain, decreasing swelling, promoting bed mobility, enhancing hip joint functional range of motion, and strength of pelvic girdle and lower limb muscles.

Long-term goals: It is intended at enhancing the full range of motions of the hip joint, maintaining the strength of the pelvic girdle, core, and lower limb muscles, increasing endurance, and assisting the patient in gradually returning to functional activities and improving his cardiovascular function.

The physiotherapy interventions given were cardiovascular and respiratory training, in-bed mobility exercises, stretching exercises for distal joint muscles, and strengthening for upper and lower extremity muscles, as described in Table 2.

**Table 2:** Therapeutic interventions

Intervention	Specification and Dosage	Rationale
Patient education	About the condition and the rehabilitation protocol	To improve the psychological status of the patient and the relatives.
Cardiovascular and respiratory training	Breathing exercises, inspiratory hold exercises	To control breathing, provide relaxation, strengthen the inspiratory muscles of respiration and improve cardiovascular endurance.
In-bed mobility exercises	Log rolling to either side, long sitting, bedside sitting, standing with and without support, walking with and without assistance.	To help the patient regain his functional activities.
Stretching exercises for distal joint muscles	Achilles’ tendon - 3 reps with 30 seconds hold	To maintain the length of the muscle.
Strengthening of upper extremity muscles	With 1 kg weight proceeding to 1.5 and 2 kg weight gradually. 10 reps	To strengthen the muscles of the arm, forearm, and hand.
Strengthening of lower extremity muscles	Isometric exercises to quadriceps, hamstrings, and back muscles – 10 reps with 10 seconds hold. Gradually progressing to isotonic exercises of quadriceps, hamstrings, and back muscles – 10 reps with 10 seconds hold.	To strengthen the muscles of the back and knee joint.

Follow-up and outcome of interventions

Visual Analog Scale (VAS): There was significant relief in pain of the patient's pre- and post-treatment from a score of 9 out of 10 to a score of 4 out of 10 on VAS.

**Figure 4:**
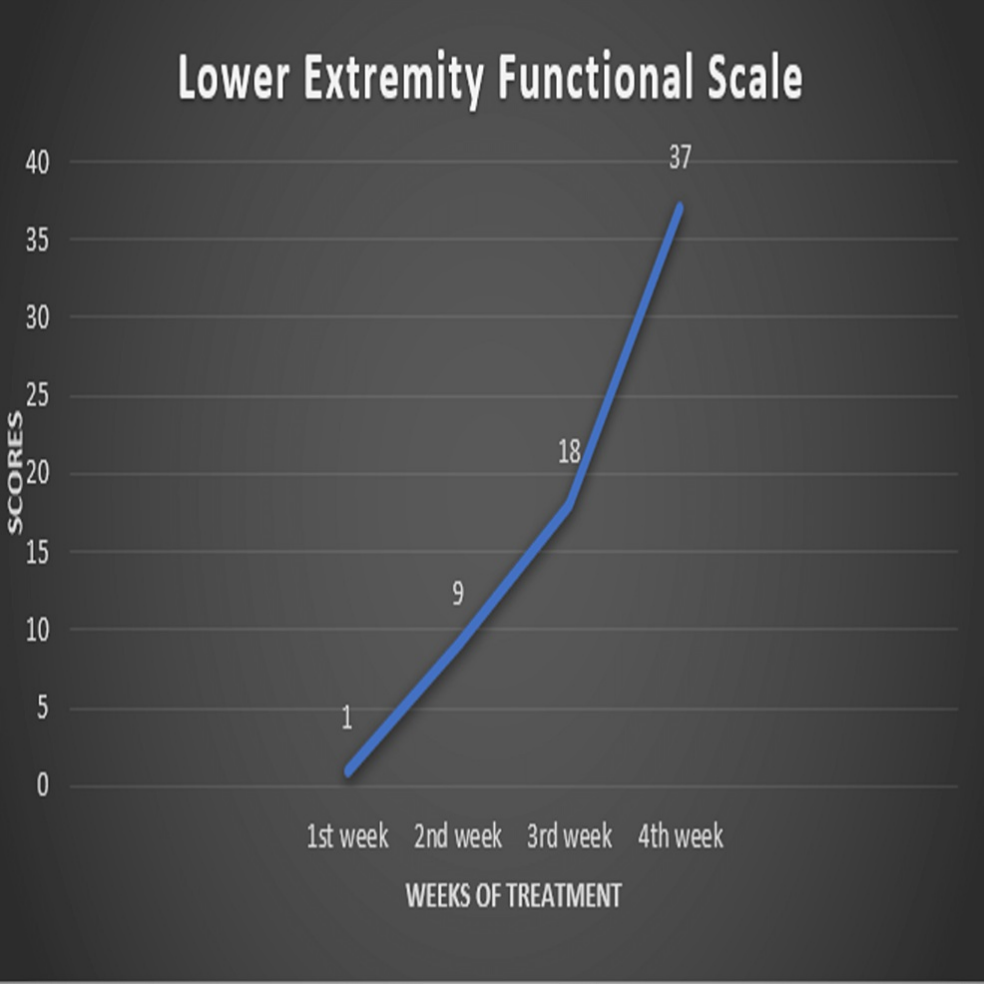
Lower Extremity Functional Scale (LEFS). The X-axis indicates the weeks of treatment. The Y-axis indicates the scores out of 80.

Lower Extremity Functional Scale (LEFS): The score improved significantly after therapy, with the recovery of various lower-limb functional activities such as rolling over the bed, sitting, standing, doing day-to-day household work, and getting in and out of the bath, walking. The lowest score before therapy was 0, and the highest score after treatment was 80, with all functional activities normal. After one month of therapy, the patient had a score of 37 (Figure 4).

European quality-of-life questionnaire-5D-5L 

The pre-treatment score, i.e., 55554, indicates extreme problems with mobility, extreme problems with self-care, extreme problems doing usual activities, severe pain or discomfort, and severe anxiety or depression.

The post-treatment score, i.e., 22332, suggests minor mobility issues, minor self-care issues, moderate difficulty doing daily tasks, moderate pain or discomfort, and minor anxiety or sadness.

## Discussion

MLL is a shearing force-induced disorder that affects the lumbar spine, scapula, thigh, knee, flank, pelvis, and greater trochanter. Traumatic situations are most frequently the cause of MLL, with vehicle accidents being the most common culprit [10]. A bacterial infection may be a worry in MLL since the mechanism of injury is often traumatic, and there is an open skin lesion [3]. They are rarely detected early, and delays in diagnosis might make management more challenging [7]. The goal of this case report was to provide a complete protocol with different step-by-step treatment strategies for the management of Morel-Lavallée syndrome in order to achieve the patient's performance goals and prognosis. It also highlighted the necessity of physiotherapy in these orthopedic conditions. This research is the first to outline the basic physiotherapy rehabilitation protocol with patient recovery results for Morel-Lavallée syndrome. The poor financial status of the patient and limited hospital stay affected the total recovery in terms of pain and physical activity, thus limiting the case report. The delays in starting the physical therapy make the condition worse and delay the return of the patient’s physical activity. Early management and physical therapy, and rehabilitation help in improving the prognosis.

## Conclusions

This study found that by employing strengthening exercises and other physical therapy interventions, there was a significant gain in joint range of motion, muscular strength, and functional independence, as well as cardiovascular and pulmonary functions after four weeks of rehabilitation. This case study highlights the importance of systematic physical rehabilitation following Morel-Lavallée syndrome to assure the patient's full recovery.
